# A step-by-step protocol for capturing conformational snapshots of ligand gated ion channels by single-particle cryo-EM

**DOI:** 10.1016/j.xpro.2022.101732

**Published:** 2022-09-30

**Authors:** Kaihua Zhang, David Julius, Yifan Cheng

**Affiliations:** 1 Department of Biochemistry and Biophysics, University of California, San Francisco, San Francisco, CA 94158, USA; 2 Department of Physiology, University of California, San Francisco, San Francisco, CA 94158, USA; 3 Howard Hughes Medical Institute, University of California, San Francisco, San Francisco, CA 94158, USA

**Keywords:** Structural biology, Cryo-EM

## Abstract

Capturing conformational snapshots by single-particle cryo-EM facilitates the analysis of ligand binding and activation mechanisms for ion channels and other receptor complexes. Here, we present a protocol to capture intermediate states of nanodisc-reconstituted TRPV1. This protocol covers sample preparation, data acquisition, and image processing with focuses on the symmetry expansion and focused 3D classification. This protocol can be adapted to different proteins and samples.

For complete details on the use and execution of this protocol, please refer to Zhang et al. (2021).

## Highlights

• Step-by-step biochemical approach on sample preparation
• Detailed steps for cryo-EM data acquisition
• Image processing procedure to capture intermediate conformational snapshots

## Abstract (editor highlights)

Publisher’s note: Undertaking any experimental protocol requires adherence to local institutional guidelines for laboratory safety and ethics.

## Graphical abstract

**Figure:**
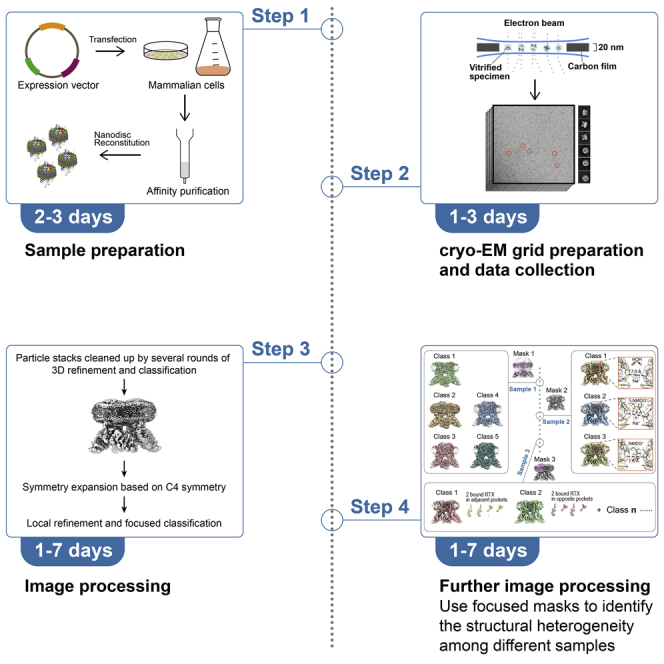

## Before you begin

We recently exploited single particle cryo-electron microscopy (cryo-EM) to capture multiple intermediate states of the capsaicin receptor, TRPV1, in various pharmacological conditions. These conformational snapshots revealed dynamic transitions of the channel associated with binding of different agonists or in the presence of different permeating cations (Zhang et al., 2021). This was accomplished by collecting cryo-EM datasets from these pharmacologically distinct samples, followed by computational processing to separate particles with distinct conformations within the same sample. The protocol outlined below begins with a description of sample preparation in which TRPV1 was complexed with an activating spider toxin (DkTx) or a potent vanilloid agonist (resiniferatoxin, RTX) in the presence of a different permeating cations, including monovalent sodium or large organic cations (NMDG/QX-314) prior to grid freezing. Procedures for single particle cryo-EM data acquisition and image processing are also described.

### Sample preparation and reagent procurement

**Timing: 2 days for protein preparation**

1. Recombinant TRPV1 protein with an N-terminal MBP-tag is expressed in HEK293 GnTI^-^ (Liao et al., 2013). Cell pellets are prepared from cell culture following these sub-steps:
  a. Cell pellets are obtained via the centrifugation at 1,000 × *g* for 10 min and gently washed in buffer 1×PBS.
  b. The samples are re-pelleted followed by discarding the supernatant, flash-frozen in liquid nitrogen, and stored at −80°C.
2. Frozen cell pellets from 300 mL culture are thawed on ice, resuspended with a Dounce homogenizer in buffer (10 mL/g wet cell pellets) containing 20 mM HEPES, pH 7.4, 150 mM NaCl, 200 μM TCEP supplemented with protease inhibitor (Sigma), followed by these sub-steps:
  a. An equal volume of 2× Membrane solubilization buffer is added to the homogenized sample and incubated at 4°C for 2 h.
  b. The supernatant fraction is then collected after centrifugation at 35,000 × *g* for 30 min.
3. For TRPV1 purification and nanodisc reconstitution:
  a. Mix the supernatant fraction with 800 μL of amylose resin and incubate at 4°C for 30–45 min.
  b. Load the mixture onto a gravity chromatography column (Bio-Rad company) and wash with Washing buffer until the concentration of non-specific proteins in the flowthrough buffer is below an empirical threshold based on the UV absorbance at 280 nm (Generally around 20 column volumes of buffer for washing).
  c. Elute TRPV1 protein from the amylose resin with around 5 column volumes of Washing buffer supplemented with 20 mM maltose.
  d. Reconstitute eluted TRPV1 protein immediately into MSP2N2-based nanodiscs by incubating the mixture at the ratio TRPV1 monomer:MSP2N2:soybean lipid = 1:1.8:80 and removing the detergents through 2–3 batches of Bio-beads SM2 (Bio-Rad), followed by size exclusion chromatography (Superdex 200 Increase 10/300 GL) in buffer A/B/C (see buffer table in Materials and equipment), respectively. (Expression plasmid of MSP2N2 scaffold protein is obtained from Addgene (Plasmid #29520).
  e. Pool fractions from the nanodisc reconstituted TRPV1 peak together for preparation of cryo-EM grids. A detailed protocol for nanodisc reconstitution can be found in (Gao et al., 2016; Zhang et al., 2021). After this step, nanodisc reconstituted channels can be fresh frozen for storage at −80°C. Troubleshooting 1.
4. Reagents, including RTX and NMDG were obtained from Sigma, and QX 314 chloride from Tocris.
5. Aliquots of purified DkTx were prepared in-house (Bohlen et al., 2010) and stored at −80°C.

**Pause point:** The toxin DkTx (∼8 kDa) are always prepared ahead of time to obtain the complex TRPV1-DkTx.

6. Quantifoil R1.2/1.3 300 mesh Au holey carbon grids were used.

***Alternatives:*** More grids can be found at the websites of the company Quantifoil or other providers such as Quantifoil R1.2/1.3 300 mesh Cu holey carbon grids, C-flat holey carbon grids and Lacey carbon grids.

## Key resources table

**Table:** 

REAGENT or RESOURCE	SOURCE	IDENTIFIER
**Chemicals, peptides, and recombinant proteins**		

Double-knot toxin (DkTx)	Bohlen et al. (2010)	N/A
Resiniferatoxin (RTX)	Sigma	Cat#R8756
N-Methyl-D-glucamine (NMDG)	Sigma	Cat#M2004
QX 314 chloride	Tocris	Cat#2313

**Deposited data**		

TRPV1, DkTx pre-bound	Zhang et al. (2021)	EMD-23139; PDB: 7L2S
TRPV1, DkTx singly-bound	Zhang et al. (2021)	EMD-23161
TRPV1, DkTx, pre-open	Zhang et al. (2021)	EMD-23138; PDB: 7L2R
TRPV1, DkTx, partial open	Zhang et al. (2021)	EMD-23140; PDB: 7L2T
TRPV1, DkTx, open	Zhang et al. (2021)	EMD-23141; PDB: 7L2U
TRPV1, RTX/NMDG a	Zhang et al. (2021)	EMD-23143; PDB: 7L2W
TRPV1, RTX/NMDG b	Zhang et al. (2021)	EMD-23142; PDB: 7L2V
TRPV1, RTX/NMDG c	Zhang et al. (2021)	EMD-23144; PDB: 7L2X
TRPV1, 1 perturbed PI	Zhang et al. (2021)	EMD-24084; PDB: 7MZ6
TRPV1, 1 partially bound RTX	Zhang et al. (2021)	EMD-24086; PDB: 7MZ9
TRPV1, 4 partially bound RTX	Zhang et al. (2021)	EMD-24085; PDB: 7MZ7
TRPV1, 2 bound RTX in adjacent pockets	Zhang et al. (2021)	EMD-24087; PDB: 7MZA
TRPV1, 2 bound RTX in opposite pockets	Zhang et al. (2021)	EMD-24091; PDB: 7MZE
TRPV1, 3 bound RTX and 1 perturbed PI	Zhang et al. (2021)	EMD-24088; PDB: 7MZB

**Software and algorithms**		

SerialEM	Mastronarde (2005)	http://bio3d.colorado.edu/SerialEM/; RRID: SCR_017293
MotionCor2	Zheng et al. (2017)	https://msg.ucsf.edu/software; RRID: SCR_016499
GCTF	Zhang (2016)	https://cam.ac.uk/kzha; RRID: SCR_016500
Relion 3.0	Zivanov et al. (2018)	https://cam.ac.uk/relion; RRID: SCR_016274
Relion 3.1	Scheres (2020)	https://cam.ac.uk/relion; RRID: SCR_016274
cryoSPARC	Punjani et al. (2017)	https://cryosparc.com; RRID: SCR_016501
cisTEM	Grant et al. (2018)	https://cistem.org/; RRID: SCR_016502
PyEM	Daniel Asarnow, Yifan Cheng Lab	https://github.com/asarnow/pyem; https://doi.org/10.5281/zenodo.3576630
UCSF Chimera	Pettersen et al. (2004)	https://www.cgl.ucsf.edu/chimera; RRID: SCR_004097

**Other**		

QUANTIFOIL R1.2/1.3 300 mesh Au holey carbon grids	Quantifoil	https://www.quantifoil.com/

## Materials and equipment

**Table:** 2× Membrane solubilization buffer

Reagent	Final concentration	Amount
1 M HEPES	100 mM HEPES	10 mL
5 M NaCl	150 mM NaCl	3 mL
10% DDM	1.5% DDM	15 mL
100 mM TCEP (fresh)	4 mM TCEP	4 mL
100% Glycerol	20% Glycerol	20 mL
milliQ water	N/A	48 mL
**Total**	**N/A**	**100 mL**

***Note:*** The buffer is stored at 4°C with pH at 7.4.

**Table:** Washing buffer

Reagent	Final concentration	Amount
1 M HEPES	20 mM HEPES	1 mL
5 M NaCl	150 mM NaCl	1.5 mL
10% DDM	0.03% DDM	0.15 mL
100 mM TCEP (fresh)	100 μM TCEP	0.05 mL
100% Glycerol	10% Glycerol	5 mL
milliQ water	N/A	42.3 mL
**Total**	**N/A**	**50 mL**

***Note:*** The buffer is stored at 4°C with pH at 7.4.

**Table:** Buffer A/B/C for gel filtration

Component	Buffer A	Buffer B	Buffer C	Final concentration
1 M HEPES	2 mL			20 mM
5 M NaCl	3 mL	N/A	100 μL	150 mM (Buffer A) /5 mM (Buffer C)
314 mM NMDG	N/A	2 mL	N/A	6.3 mM (Buffer B)
100 mM TCEP (fresh)	100 μL			100 μM
15% NaOH	to pH 7.4	to pH 7.1	to pH 7.3	5.6 mM (Buffer A) /375 μM (Buffer B) /6 mM (Buffer C)
milliQ water	to 100 mL	to 100 mL	to 100 mL

***Note:*** We didn’t test the maximum storage of these buffers. It is recommended that buffer A/B/C should be freshly prepared and stored at 4°C, particularly for the component TCEP.

• PELCO easiGLOW Glow Discharge Cleaning System (Ted Pella).

***Alternatives:*** EMS 700 glow discharge (Electron Microscopy Sciences).

• Vitrobot Mark IV (Thermo Fisher Scientific).

***Alternatives:*** EM GP2 automatic plunge freezer (Leica Microsystems).

• Titan Krios microscope (Thermo Fisher Scientific FEI) operated at 300 kV and equipped with a K3 direct electron detector (Gatan).

***Alternatives:*** Cryo-EM data in this study were collected using a 300kV Titan Krios microscope (Thermo Fisher Scientific FEI), equipped with a K3 camera from Gatan Inc. Alternatively, it is possible to use cryogenic electron microscopes operating at 200kV, equipped with FEG and a direct detection camera, such as Talos Arctica or Glacios (Thermo Fisher Scientific FEI). In terms of the camera, equivalent direct electron detection cameras from other manufactures are also suitable; In addition, 300 kV or 200 kV microscopes from JEOL, such as cryoARM, are also suitable instrument.

• Computational resources and software required for image processing are listed in the key resources table.

***Note:*** In our study, the computational resources being used include UCSF Wynton high-performance compute (HPC) cluster (https://wynton.ucsf.edu/hpc/) and stand-alone GPU workstations within Yifan Cheng laboratory. The stand-alone GPU workstations are configured with the linux system CentOS 7 and the following hardware: earlier version: CPU (Intel Xeon E5 v4 2.2 up to 3.1 GHz 10-Core Processor), CPU Memory (2 × Crucial 64 GB Quad Channel Registered ECC DDR4 at speeds up to 2133 MT/s), Storage (1 TB SATA III MLC SSD; 18 TB HDD), GPU (4 × GeForce GTX 1080 Ti FE with 3584 CUDA Cores and 11 GB graphic ram); upgraded version: CPU (EPYC 73F3 3.5GHz 16-Core Procs), CPU Memory (512GB DDR4-3200), Storage (1.92 TB U.2 NVMe SSD plus HDD), GPU (A40 with 48GB GPU memory). For individual programs, including Relion, cisTEM and cryoSPARC, optimized hardware configuration can be found in the respective websites.

## Step-by-step method details

### Complex preparation right before freezing cryo-EM grids

**Timing: 30 min**

Expression and purification of TRPV1 in HEK293 cells, and reconstitution of purified TRPV1 into lipid nanodisc was performed following procedures described above. Typically, we will use negative-stain EM to assess the quality of nanodisc reconstituted TRPV1. A typical protein preparation from around 300 mL of cell culture produces 0.5–2 mg of purified and nanodisc reconstituted TRPV1 protein that can be divided into multiple aliquots for separate rounds of cryo-EM grid preparation, either to optimize conditions for freezing cryo-EM grids or for preparing complexes with different ligands or other factors. Such additives were usually mixed with aliquoted proteins prior to depositing on cryo-EM grids.

1. Incubate nanodisc reconstituted TRPV1 in buffer A with DkTx at the molar ratio of 1:3.5 and the final concentration of DkTx at 20 μM for 20–30 min at ∼24°C. This sample is referred to as TRPV1-DkTx.

***Note:*** It is known that two DkTx molecules bind to one tetrameric TRPV1 protein.

2. Incubate nanodisc reconstituted tetrameric TRPV1 in buffer B with RTX at a final concentration of 40–55 μM (molar ratio of 1:6.9–9.5) supplemented with QX-314 and NMDG at ∼24°C.

***Note:*** The final concentration of QX-314 and NMDG was 28 mM and 23 mM, respectively.

***Note:*** This sample is referred to as TRPV1-RTX/NMDG.

***Note:*** Each tetrameric TRPV1 protein has four binding sites for the agonist RTX. RTX reportedly activates wild-type rat TRPV1 with an EC_50_ of ∼75 pM (Johnson et al., 2006). Permeation by QX-314 or NMDG by activated TRPV1 has been described previously (Jara-Oseguera et al., 2019).

3. Incubate nanodisc reconstituted TRPV1 in buffer C with RTX at final concentration of 40–55 μM (molar ratio of 1:6.9–9.5) supplemented with QX-314 at ∼24°C. The final concentration of QX-314 and Na^+^ was 25 mM and 9 mM, respectively. This sample is referred to as TRPV1-RTX/lowSodium.

***Note:*** We purposely used a low concentration of Na^+^ in the final buffer recipe to favor permeation by the large organic cation QX-314.

**CRITICAL:** Avoid multiple freeze-thaw cycles or always use freshly prepared protein/peptide. In the case of TRPV1, aliquotes with only one freeze-thaw cycle behaved similarly to freshly prepared samples. We suggest that, prior to preparation of complexes, the Vitrobot Mark IV is made ready for use, including preparation of liquid ethane and glow-discharged grids, filter paper replacement, filling of humidifier, etc.

### Cryo-EM grid preparation

**Timing: 1–3 h**

These steps describe preparation of the Vitrobot Mark IV and grids for freezing in preparation for loading into microscopes.

4. Turn on the Vitrobot by switching button at the back of instrument and wait until Main User interface becomes operational.
5. Fill the humidifier with distilled water and mount it by following the standard instructions.

***Note:*** Be careful to mount and remove the humidifier. Make sure that the sealing O-ring is on the top of humidifier. Empirically, 30 mL of water should be enough for filling.

6. Set chamber temperature to 20°C. Increase humidity to 100% and turn on device. Ideally, equilibrate the chamber and Vitrobot for approximately 30 min.

***Optional:*** Temperature and humidity settings may vary in specific cases, such as when working on thermo-sensitive samples.

7. Install new blot papers (Whatman no. 1) on the pad pair a few minutes before first freezing the first grid. Make sure the sharp tip of the Vitrobot tweezer (Ted Pella) works well before using it to handle grids.

***Note:*** The effectiveness of blotting filter papers decreases after extended exposure to high humility. Furthermore, each blotting paper is usually good for only 8 blotting. Thus, the blotting filter paper should be replaced accordingly to prevent contamination and wetting in high humility.

8. Assemble and cool the cryogen container by filling the reservoir with liquid nitrogen (LN) and placing a small amount in the copper cup.
  a. Place 4-slot grid box(es) into the metal stand supporting up to 4 grid boxes.
  b. Allow LN in the reservoir to equilibrate and wait until LN in the copper cup stops bubbling.
  c. Fill the copper cup with ethane gas (Airgas company) slowly.
    ***Note:*** Ethane gas liquifies when encountering cold copper cub and clear liquid ethane accumulates in the copper cub.
  d. Turn off ethane gas when the copper cup is full of liquid ethane.
  e. Remove the metal spider when ethane begins turning from clear to white.
    **CRITICAL:** Ethane is flammable and may form an explosive gas. Thus, keep it away from heat and fire. When disposing of liquid ethane, pour it into a large quantity of liquid nitrogen slowly. Rapid pouring of liquid ethane may cause splashing (with a loud bang). Liquid ethane causes burning pain on contact with skin. Always wear eye protection and prop PPE when handling liquid ethane!
    ***Note:*** This course for cooling the cryogen apparatus is also available at this link: https://emcore.ucsf.edu/content/vitrobot-markiv-notes.

9. Transfer gold grids covered with holey carbon film (Quantifoil, 300 mesh 1.2/1.3) from storage box to grid holder with holey carbon side facing up.
  a. Gently place the loaded grid holder onto the sample table of PELCO easiGLOW equipment (Ted Pella).
  b. Put the bell jar back on the seal correctly.
  c. On the screen, adjust the settings to 30 s at 30 mA and press “AUTO RUN” to start the process.
  d. Wait until the process finishes normally and check to see if the vented system goes back to atmospheric pressure.
  e. Carefully remove the grid holder and save in a clean petri dish.

***Note:*** We use a glass slide as grid holder for glow discharge. Detailed information, including program modification, can be found at https://emcore.ucsf.edu/content/easiglow-protocol.

***Optional:*** The glow-discharge setting may be optimized for different samples.

10. Pick up one glow-discharged grid with the Vitrobot tweezer and properly mount it to the metal rod.
11. Operate Vitrobot to lift ethane container and move tweezer to lower position in the chamber.
12. Carefully apply 3 μL of sample solution onto the holey-carbon side.
13. Blotting is done with force set to 2 and time for 3.5 s, followed by automatic plunge freezing.

***Note:*** This blotting force and time generally works well for our samples. For different samples, one may need to optimize the settings to find optimal condition for vitreous ice, which is often necessary when working with new samples.

14. Place the frozen grid into the grid box in the cryogen reservoir. Troubleshooting 2.

***Note:*** With practice, grid transfer will occur smoothly and without bending the grid. The grid should be submerged in liquid nitrogen to prevent crystallization or melting of vitreous ice.

15. Repeat steps 10–14 to freeze more grids if needed.

***Note:*** Typical grid boxes have 4 slots, one for each cryo-grid. Make sure to mark these slots in advance so that the freezing condition of each grid can be recorded properly.

16. Transfer the frozen grids into a storage dewar filled with LN for subsequent grid screening and data collection.

***Note:*** Make sure that the storage dewar is always filled with sufficient LN.

**Pause point:** The grids are generally frozen ahead of microscope time. The time lag may vary between grid freezing and data collection.

### Cryo-data collection

**Timing: 1–3 days**

The steps in this section are designed for screening grid quality and acquiring images for data processing. Troubleshooting 3.

17. Cool the transfer station, assembly tools, and autoloader cassette in LN.
18. Carefully clip each grid with a clip ring and C-clip and transfer to pre-cooled autoloader cassette.

***Note:*** More information about grid clipping can be found at https://cryo-em-course.caltech.edu/.

19. Properly insert well-clipped grids into cassette and load into the autoloader of the microscope (Titan Krios).

***Note:*** Wait until the inventory updates and the microscope is ready for grid screening.

20. Through the software SerialEM (Mastronarde, 2005), screen the loaded grids individually and mark the qualified grids for subsequent data acquisition, including particle dispersion and ice thickness.

***Alternatives:*** The software EPU can also be used for data collection.

***Optional:*** For each stage movement, we typically collect images from 9 holes by using image shift coupled with beam tilt. With fringe-free illumination (Weis and Hagen, 2020) setup, we collect 3 or 4 images per hole. Following this strategy, more than 5,000 movie stacks can be recorded per day.

***Note:*** All datasets were obtained in the super-resolution mode, including samples of TRPV1-DkTx, TRPV1-RTX/NMDG and TRPV1-RTX (low Sodium). The raw movies were saved in compressed tiff format after subtraction with dark reference. Detailed information is provided in Table 1.

### Image processing

**Timing: Variable from a few days to weeks**

The steps in this section are aimed at obtaining 3D reconstructions of protein samples through data processing. There are multiple programs available such as Relion, cisTEM, and cryoSPARC.

21. The first step in image processing is to correct beam induced image motion.

***Note:*** We use MotionCor2 (Zheng et al., 2017) to perform on-the-fly motion correction through the software Scipion (de la Rosa-Trevin et al., 2016) once an image is collected as a movie stack. Normally, all motion corrected images are ready for further processing soon after data acquisition is completed.

***Optional:*** Motion correction can also be performed after data acquisition and/or using similar algorithms implemented in other programs, such as cryoSPARC or Relion.

***Note:*** The common parameters needed for all image processing pipelines include pixel size, accelerating voltage, spherical aberration, and total exposure dose. Further introduction to the use of cryoSPARC can be found at https://guide.cryosparc.com/.

22. The next step is to determine defocus or contrast transfer function (CTF) of each image.
  a. Motion corrected images in MRC format are imported to cryoSPARC (Punjani et al., 2017).
  b. Select “Patch CTF Estimation (multi)” in the Job Builder and add imported micrographs as input.

***Note:*** Make sure the amplitude contrast and other default values are pertinent.

***Optional:*** CTF estimation can be performed using GCTF or other software.

23. Manually assess micrograph quality by enabling “Manually Curate Exposures” in the Job Builder and rejecting inferior micrographs based on CTF estimation results.

***Note:*** The criterion for rejection is subjective. We keep the micrographs for further analysis by setting a threshold for the CTF fitting up to a certain resolution (e.g., 6 Å) at highest frequency.

24. Clone the job created at step 23 and drag and drop the accepted micrographs from last step as input. Randomly select 100 micrographs and submit the job.

***Note:*** This set of micrographs will be used for blob picking of particles and 2D templates selection.

***Optional:*** 100 micrographs can be randomly selected using “Exposure Sets job.”

25. Measure the size of the protein particles at different views and examine a range of minimum and maximum particle diameters in the job “Blob picker”. Monitor the Blob picking and complete the job with optimal parameters.

***Note:*** e2display.py in EMAN2 package can be used for diameter measurement.

26. Extract the particles by using the proper box size and cropping with 4 times at Fourier space.

***Note:*** Down-scaling particle stacks is one option to speed up data processing. Recommendations regarding box size can be found at https://blake.bcm.edu/emanwiki/EMAN2/BoxSize.

27. Move to “2D classification” in Job Builder with extracted particles as input. Set “Number of 2D classes”, between 20 and 50 depending on the number of extracted particles in the stack.
28. Once 2D classification job finishes, execute “Select 2D” job to select the representative 2D projections for template creation.

***Optional:*** 2D projections for template-based picking can also be created from atomic structure if any similar structure is available in the PDB database.

29. Drag and drop the selected micrographs at step 23 and 2D templates from representative 2D averages at step 28 into the active “Template picker” job with proper particle diameter set.

***Optional:*** Apart from Blob picker and Template picker, it is worthy trying “Deep Picker” powered by network training in some tricky cases.

30. Wait until completion of particles picking on all gratified micrographs from step 29, and repeat steps 26 and 27 to perform particles extraction and 2D classification.
31. Submit “Select 2D″ job to retain the particles from acceptable classes. Troubleshooting 4.

***Note:*** In general, during 2D classification, we only discard particles from classes that are obviously junk. Further clean-up is performed in the following 3D classification. This helps to preserve particles of different conformations.

32. The selected particles are further analyzed with 3D classification in cryoSPARC. The “Ab-initio Reconstruction” job is used to generate multiple references, followed by “Heterogenous Refinement” job.

***Note:*** To speed up the process for large dataset, one can either interrupt the “Ab-initio Reconstruction” process, once a satisfactory result is obtained, or limit the number of particles per classes (such as 10,000 particles per class). The particles selection at step 31 can be tightened up to generate reasonable references using high-scored particles. This 3D classification step should roughly discriminate between good classes and junk classes without consideration of high-resolution information.

33. Save the particles within good classes and convert the cryoSPARC-derived cs file to the star file, which can be recognized by the software Relion (Scheres, 2020; Zivanov et al., 2018).

***Note:*** The file conversion between cs and star files was performed using the command csparc2star.py in the package UCSF pyem (https://zenodo.org/record/3576630). Make sure to enable the argument “--swapxy”.

34. Run the “Particle extraction” job to extract the particles using the coordinates file generated by cryoSPARC.

***Note:*** Depending on the number of particles in the dataset, it is worth downscaling the particle stacks by a factor of 2 for subsequent reconstructions to speed up data processing, as we did in our data sets.

35. The extracted particles were subjected to the “3D classification” job in Relion (classes: 9; iterations: 35) with imposing C4 symmetry and using the 3D reconstruction from cryoSPARC as the initial reference.

***Note:*** If the reconstructed maps are available from the same protein or other members of the same family, then they can technically be used as initial references.

36. Launch the ‘‘Subset selection’’ job to prepare the star files of each class with reasonable protein features or combined classes with the same conformation after 3D classification separately.
37. These star files were used for particle extraction at the original pixel level. Another round of 3D classification with imposing C4 symmetry was performed per class to further clean up the particles and identify sub-states.

**Table 1:** Data collection statistics

Samples	Total images	Pixel size (Å/pix)	Dose rate (e^-^/pix/s)	Exposure time (s)	Frames per image
TRPV1-DkTx	7858 + 15288	0.8488	10.0	5	100
TRPV1-RTX/NMDG	3194	0.835	8.0	6	120
TRPV1-RTX (lowSodium)	2427	0.834	8.0	6	120

**Figure 1:**
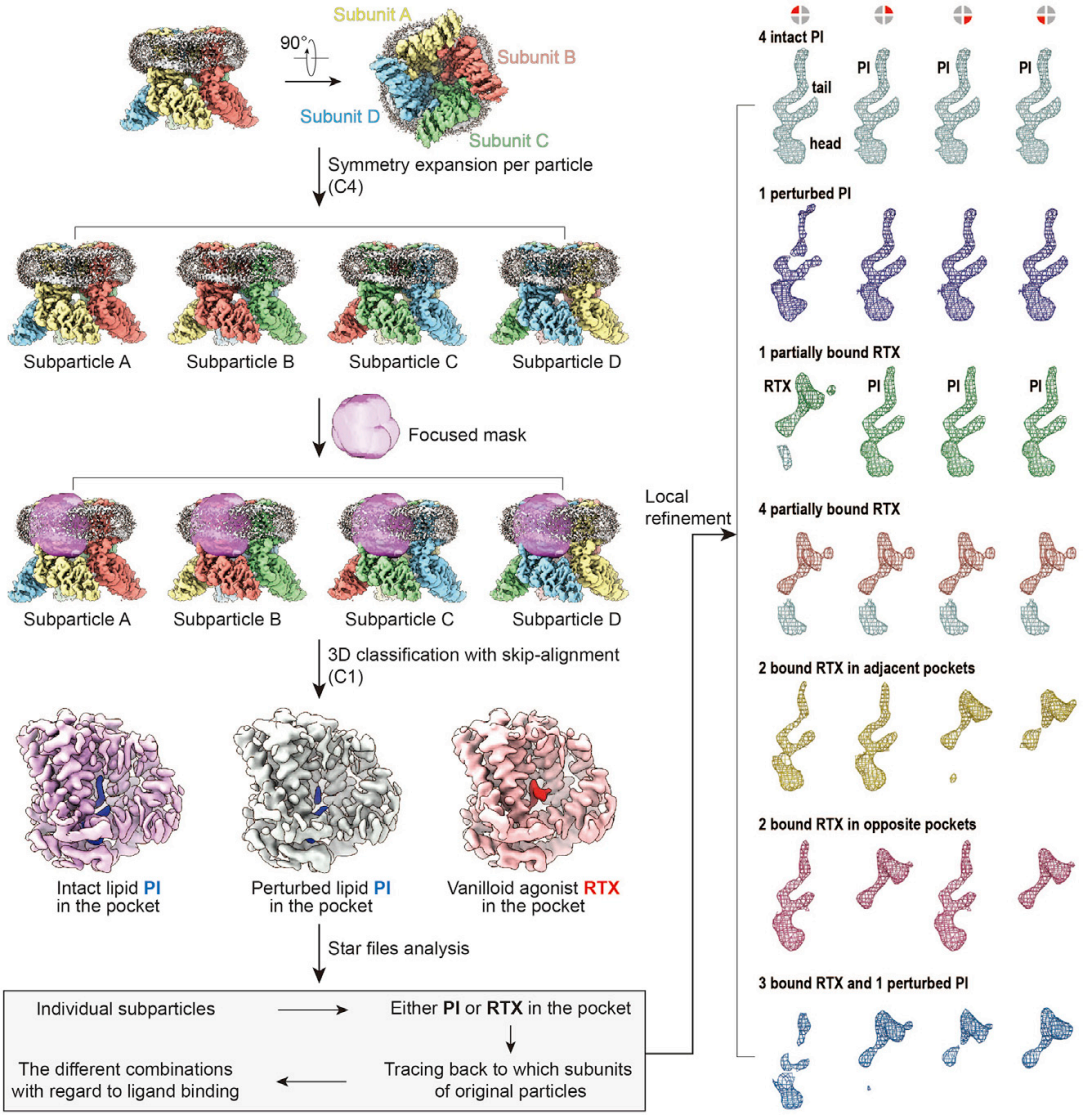
A flow chart illustrating the procedure of using symmetry expansion and focused classification to identify stoichiometry of RTX binding to TRPV1 This procedure was applied to the TRPV1 RTX/lowSodium sample and allowing capturing of different intermediate binding states of RTX in tetrameric TRPV1.

The steps described above cover routine procedures in determining structures of a tetrameric ion channel. In the following, we describe procedures specific to isolating various intermediate states via symmetry expansion and/or focused classification. A flowchart illustrating these procedures is shown in Figure 1.

38. To better identify heterogeneity among each subunit of a particle, we performed symmetry expansion using the command “relion_particle_symmetry_expand” in Relion. Troubleshooting 5.

***Note:*** During symmetry expansion, each particle is assigned four different sets of Euler angles so that each assignment rotates the particle by 90° around the C4 symmetry axis. Thus, the total number of particles in the dataset was quadrupled.

39. The quadrupled particle stacks were subjected to local refinement and focused classification using a 3D mask around the region of interest without imposing symmetry (Regularization parameter T=20; skip-align). Again, particles of poor classes were removed.
40. To further classify different binding modes of DkTx, stoichiometric binding of RTX, or NMDG-induced dilation of the selectivity filter, we performed another round of focused 3D classification with the respective mask focusing on the targeted region.

***Note:*** For DkTx-containing samples, the mask covered DkTx density in the outer pore region of the symmetry expanded tetramer.

***Note:*** For TRPV1-RTX/NMDG sample, the mask covered the pore region of the symmetry expanded tetramer.

***Note:*** For the TRPV1 RTX/low-sodium sample, the mask covered the vanilloid binding pocket.

***Note:*** This focused classification of symmetry expanded particles revealed multiple substates in each sample. This procedure is also described in the Graphical abstract.

***Note:*** The regularization parameter needs be optimized depending on the masked local regions. These focus masks were created by generating the focused maps in Chimera (Pettersen et al., 2004) and running the job “Mask creation” in Relion (extend binary map: 6 pixels; add a soft-edge: 5 pixels).

41. For the TRPV1-DkTx sample, we removed particles of poor classes after the focused classification. The remaining particles were subjected to another round of 3D classification (T=20; skip-align) without applying a focused mask and symmetry. Classes of the intact particles with different orientations of DkTx were separated during this step. For each original particle, the orientational parameter of only one expansion belonging to the same DkTx bound class was retained and used to calculate a final reconstruction.

For the TRPV1-RTX/NMDG sample, we tested multiple classification parameters, including different reference maps, initial low-pass filter, number of classes, and Regularization parameter T, for the focused 3D classification described above and selected the best classes. Any particles classified into more than one class were removed to ensure that all classes contain unique particles, with the rationale that any particle classified into more than one classes is ambiguous and thus removed from further processing. This procedure produced three final 3D reconstructions in different intermediate states.

For the TRPV1 RTX/lowSodium sample, three classes of expanded particles were characterized during 3D classification, including intact phosphatidylinositol lipid (PI), perturbed PI, or RTX in the vanilloid pocket. Based on the star files for each class, which contains information of which original particle each expanded particle belongs to, we traced each symmetry expanded particle back to its original particle, grouped all particles with the same RTX binding stoichiometry, and calculated final 3D reconstructions with specific RTX binding stoichiometry (Figure 1).

42. Particles from the different states in each sample were subjected to a further auto-refinement job “Refine3D” with local search in Relion (Initial angular sampling: 1.8; Local searches from auto-sample: 1.8).
43. “Post-processing” jobs for each reconstruction were conducted to obtain sharpened maps.

***Note:*** The nominal resolution was estimated from the averaged FSC using FSC = 0.143 criterion by calculating Directional Fourier Shell Correlation (dFSC) curves.

## Expected outcomes

In this step-by-step protocol, using the TRPV1 ion channel as an example, we have presented a strategy for separating and analyzing sub-states from three heterogenous samples. We mainly focused on using two software packages, cryoSPARC and Relion, for data processing. cryoSPARC was used to perform the initial steps of image processing, such as CTF estimation, particle picking, 2D classification, and initial 3D classification. Relion was used for further 3D classification, symmetry expansion and regrouping of particles. Using the procedures described here, we succeeded in identifying multiple intermediate conformations in all three samples. The detailed results were presented in Zhang et al. (2021).

## Quantification and statistical analysis

All the data sets were processed using motioncor2, cryoSPARC, and Relion, facilitated by scripts included in UCSF pyem. The nominal resolution was estimated following the averaged FSC based on dFSC curves using FSC = 0.143 criterion.

## Limitations

The ability to identify intermediate states from a dataset containing conformationally heterogenous particles depends on many factors, including image quality, signal-to-noise ratio (SNR) of particle images, affinity of ligands, as well as subtle differences caused by ligand binding. Furthermore, it is worth noting that achieving a reconstruction with a higher resolution and extracting conformational intermediates from a heterogeneous dataset often requires different image processing strategies. It is possible that particles contributing to different conformational intermediates are discarded if the goal is to achieve a higher resolution.

## Troubleshooting

### Problem 1

Failure to obtain the well-behaved complexes of TRPV1 with DkTx or RTX, referring to step 3 of sample preparation and reagent procurement section.

### Potential solution

It is suggested that the samples should be flash frozen and stored at −80°C until use. If possible, always prepare fresh samples for freezing and check behavior of samples first via negative-stain EM. Follow the same procedures and buffer conditions when performing sample preparation.

### Problem 2

Ice contamination on the cryo-grids or unsatisfactory ice thickness of cryo-samples, referring to steps 10–14 of step-by-step method details section.

### Potential solution

Be careful and gentle when transferring and glow-discharging grids. Avoid any contamination from touching or breathing on surfaces. Always use clean liquid nitrogen for cooling and freezing. If necessary, freeze several grids for the same sample while fine-tuning parameters and replacing blotting papers regularly.

### Problem 3

Lower quality of collected images, referring to steps 17–20 of step-by-step method details section.

### Potential solution

Make sure to prepare and freeze the samples consistently. Check the beam of the microscope and make sure microscope behavior is as expected. Always monitor data quality by analyzing micrographs as data become available during the process. Currently, cryoSPARC and Relion provide live versions for this purpose.

### Problem 4

Lower-resolution reconstructions of vitrified samples, referring to step 31 of step-by-step method details section.

### Potential solution

Make sure to prepare the well-behaved samples and collect the data sets carefully. Be cautious to discard the particles during 2D/3D classifications. Perform the 3D classification with the optimal parameters and strategies. The reconstructions of some samples may work better using certain programs.

### Problem 5

Failure to identity the heterogenous conformations of biological samples, referring to step 38 of step-by-step method details section.

### Potential solution

Attempt to fish out more particles through 3D classification from the classes with relatively limited resolution in earlier rounds of 3D classifications. Follow the up-to-date technical developments regarding data processing and play with different strategies and programs to distinguish the different states.

## Resource availability

### Lead contact

Further information and requests for resources and reagents should be directed to and will be fulfilled by the lead contact, Yifan Cheng (yifan.cheng@ucsf.edu).

### Materials availability

This study did not generate new reagents.

## Data Availability

The cryo-EM density maps were deposited the Electron Microscopy Data Bank as described in (Zhang et al., 2021). Other data are available from the corresponding author upon reasonable request.
